# Transport properties of two finite armchair graphene nanoribbons

**DOI:** 10.1186/1556-276X-8-1

**Published:** 2013-01-02

**Authors:** Luis Rosales, Jhon W González

**Affiliations:** 1 Departamento de Física, Universidad Técnica Federico Santa María, P.O. Box 110V, Valparaíso, 2390123, Chile; 2 International Iberian Nanotechnology Laboratory, Av. Mestre José Veiga, Braga, 4715-330, Portugal

**Keywords:** Graphene nanostructures, Transport properties, Magnetic field effects

## Abstract

In this work, we present a theoretical study of the transport properties of two finite and parallel armchair graphene nanoribbons connected to two semi-infinite leads of the same material. Using a single *Π*-band tight binding Hamiltonian and based on Green’s function formalisms within a real space renormalization techniques, we have calculated the density of states and the conductance of these systems considering the effects of the geometric confinement and the presence of a uniform magnetic field applied perpendicularly to the heterostructure. Our results exhibit a resonant tunneling behaviour and periodic modulations of the transport properties as a function of the geometry of the considered conductors and as a function of the magnetic flux that crosses the heterostructure. We have observed Aharonov-Bohm type of interference representing by periodic metal-semiconductor transitions in the DOS and conductance curves of the nanostructures.

## Background

Graphene is a single layer of carbon atoms ordered in a two-dimensional hexagonal lattice. In the literature, it is possible to find different experimental techniques in order to obtain graphene such as mechanical peeling, epitaxial growth or assembled by atomic manipulation of carbon monoxide molecules over a conventional two-dimensional electron system at a copper surface [1-4]. The physical properties of this crystal have been studied over the last 70 years; however, the recent experimental breakthroughs have revealed that there are still a lot of open questions, such as time-dependent transport properties of graphene-based heterostructures, the thermoelectric and thermal transport properties of graphene-based systems in the presence of external perturbations, the thermal transport properties of graphene under time-dependent gradients of temperatures, etc.

On the other hand, graphene nanoribbons (GNRs) are quasi one-dimensional systems based on graphene which can be obtained by different experimental techniques [5-8]. The electronic behaviour of these nanostructures is determined by their geometric confinement which allows the observation of quantum effects. The controlled manipulation of these effects, by applying external perturbations to the nanostructures or by modifying the geometrical confinement [9-13], could be used to develop new technological applications, such as graphene-based composite materials [14], molecular sensor devices [15-17] and nanotransistors [18].

One important aspect of the transport properties of these quasi one-dimensional systems is the resonant tunneling behaviour which, for certain configurations of conductors or external perturbations, appears into the system. It is has been reported that in S- and U-shaped ribbons, and due to quasi-bound states present in the heterostructure, it is possible to obtain a rich structure of resonant tunneling peaks by tuning through the modification of the geometrical confinement of the heterostructure [19]. Another way to obtain resonant tunneling in graphene is considering a nanoring structure in the presence of external magnetic field. It has been reported that these annular structures present resonance in the conductance at defined energies, which can be tuned by gate potentials, the intensity of the magnetic field or by modifying their geometry [20]. From the experimental side, the literature shows the possibility of modulating the transport response as a function of the intensity of the external magnetic field. In some configuration of gate potential applied to the rings, it has been observed that the Aharonov-Bohm oscillations have good resolution [21-23].

**Figure 1:**
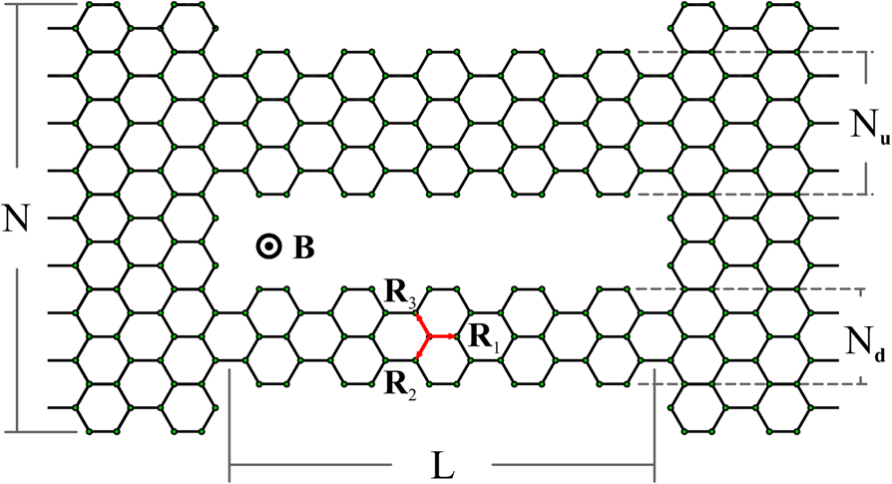
**Schematic view of the conductor.** Two finite armchair graphene ribbons (red lines). The length *L* of the conductor is measured in unitary cell units.

In this context, in this work, we present a theoretical study of the transport properties of GNR-based conductors composed of two finite and parallel armchair nanoribbons (A-GNRs) of widths *N*_*d*_ and *N*_*u*_, and length *L* (measured in unit cell units), connected to two semi-infinite contacts of width *N* made of the same material. We have thought this system as two parallel ‘wires’ connected to the same reservoirs, whether the the leads are made of graphene or another material. This consideration allows us to study the transport of a hypothetical circuit made of graphene ‘wires’ in different scenarios. A schematic view of a considered system is shown in Figure 1. We have focused our analysis on the electronic transport modulations due to the geometric confinement and the presence of an external magnetic field. In this sense, we have studied the transport response due to variations of the length and widths of the central ribbons, considering symmetric and asymmetric configurations. We have obtained interference effects at low energies due to the extra spatial confinement, which is manifested by the apparition of resonant states at this energy range, and consequently, a resonant tunneling behaviour in the conductance curves. On the other hand, we have considered the interaction of electrons with a uniform external magnetic field applied perpendicular to the heterostructure. We have observed periodic modulations of the transport properties as function of the external field, obtaining metal-semiconductor transitions as function of the magnetic flux.

## Methods

All considered systems have been described using a single *Π*-band tight binding Hamiltonian, taking into account only the nearest neighbour interactions with a hopping *γ*_0 _= 2.75*eV*[24]. We have described the heterostructures using surface Green’s function formalism within a renormalization scheme [16,17,25]. In the linear response approach, the conductance is calculated using the Landauer formula. In terms of the conductor Green’s function, it can be written as [26]:

(1) $$G=\frac{2e^{2}}{h}\bar{T}\left(E\right)=\frac{2e^{2}}{h}\text{Tr}\left[\Gamma_{L}G_{C}^{R}\Gamma_{R}G_{C}^{A}\right],$$

where $\bar{T}\left(E\right)$, is the transmission function of an electron crossing the conductor region, $\Gamma_{L/R}=i[\Sigma_{L/R}-\Sigma_{L/R}^{†}]$ is the coupling between the conductor and the respective lead, given in terms of the self-energy of each lead: *Σ*_*L*/*R*_=* V*_*C*,*L*/*R*_*g*_*L*/*R*_*V*_*L*/*R*,*C*_. Here, *V*_*C*,*L*/*R*_ are the coupling matrix elements and *g*_*L*/*R *_is the surface Green’s function of the corresponding lead [16]. The retarded (advanced) conductor Green’s function is determined by [26]: $G_{C}^{R,A}={\left[E-H_{C}-\Sigma_{L}^{R,A}-\Sigma_{R}^{R,A}\right]}^{-1}$, where *H*_*C*_ is the hamiltonian of the conductor. Finally, the magnetic field is included by the Peierls phase approximation [27-31]. In this scheme, the magnetic field changes the unperturbed hopping integral $\gamma_{n,m}^{0}$ to $\gamma_{n,m}^{B}=\gamma_{n,m}^{0}e^{2\Pi i\Delta \Phi_{n,m}}$, where the phase factor is determined by a line integral of the vector potential **A** by:

(2) $$\Delta \Phi_{n,m}=\frac{e}{h}\overset{R_{m}}{\underset{R_{n}}{\int }}dl\cdot A.$$

Using the vectors exhibited in Figure 1, **R**_1 _= (1,0)*a*, $R_{2}=-\left(1,\sqrt{3}\right)a/2$ and $R_{3}=\left(-1,\sqrt{3}\right)a/2$, where *a *= |**R**_*n*,*m*_| = 1.42 Å, the phase factors for the armchair configuration in the Landau gauge **A **= (0,*Bx*) are given by:

(3) $$\begin{array}{l} \Delta \Phi_{n,m}\left(R_{n,m}=R_{1}\right)=-\text{aB}y_{n},\\ \Delta \Phi_{n,m}\left(R_{n,m}=R_{2}\right)=-\frac{\text{aB}}{2}\left(y_{n}+\frac{a\sqrt{3}}{4}\right),\\ \Delta \Phi_{n,m}\left(R_{n,m}=R_{3}\right)=\frac{\text{aB}}{2}\left(y_{n}+\frac{a\sqrt{3}}{4}\right), \end{array}$$

where *y*_*n *_is the carbon atom position in the transverse direction of the ribbons. In what follows, the Fermi energy is taken as the zero energy level, and all energies are written in units of *γ*_0_.

## Results and discussion

### Unperturbed systems

**Figure 2:**
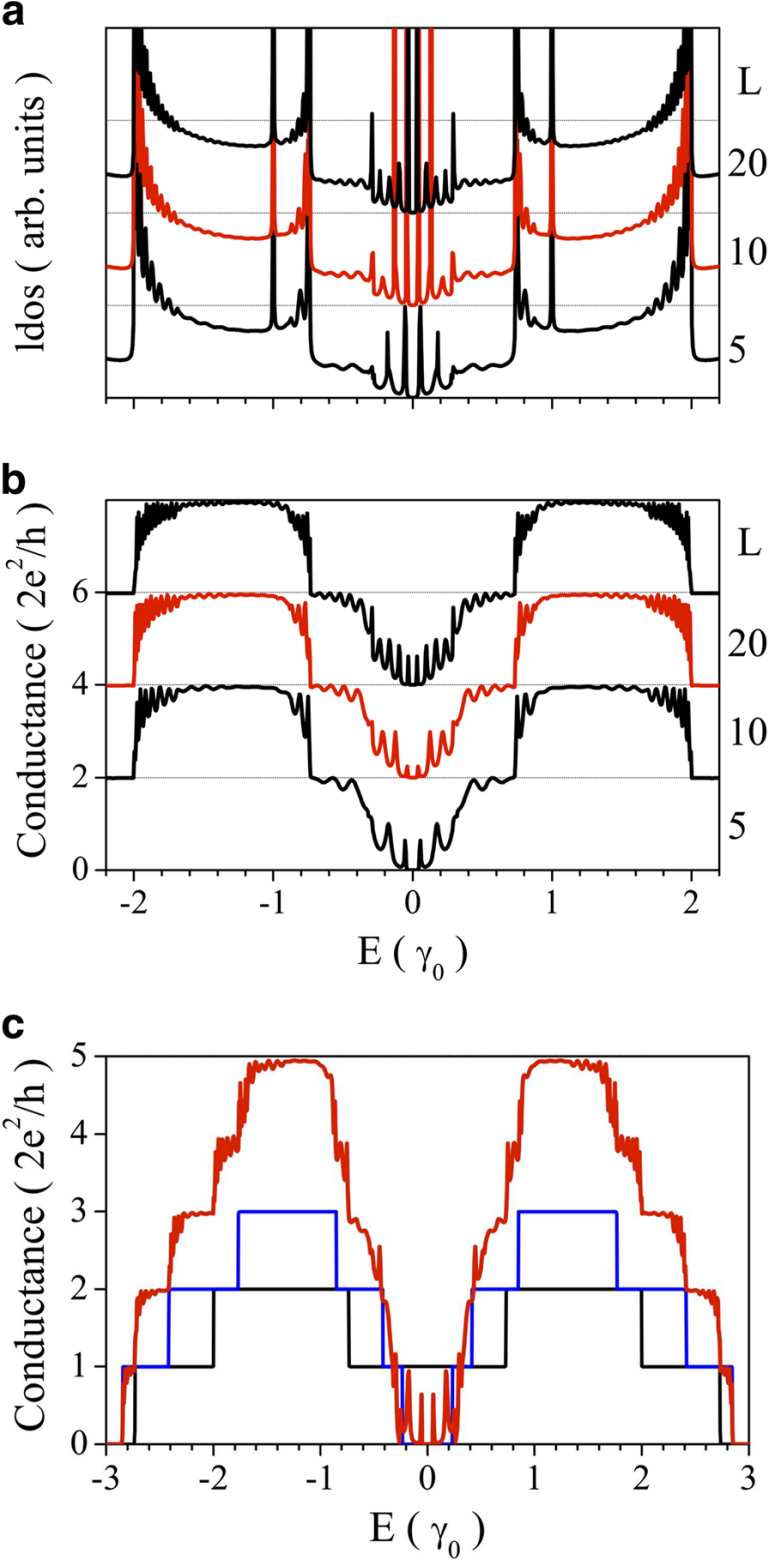
**LDOS and conductance for different geometries.** (**a**) LDOS (black line) and (**b**) conductance of two A-GRNs (red line) of widths *N*_*d *_= *N*_*u *_= 5, connected to two leads of widths *N *= 17 for different conductor lengths: *L *= 5,10,20 u.c. (**c**) Conductance of a system composed of two parallel *N*_*d *_= 5 and *N*_*u *_= 7 A-GNRs of lengths *L *= 15. As a comparison, we have included the pristine cases (black and blue curves, respectively).

Let us begin the analysis by considering the effects of the geometrical confinement. In Figure 2, we present results of (a) Local density of sates (LDOS) and (b) conductance for a conductor composed of two A-GNRs of widths *N*_*d *_=* N*_*u *_= 5 connected to two leads of width *N *= 17 for different conductor lengths (*L* = 5,10 and 20 unit cells). The most evident result is reflected in the LDOS curves at energies near the Fermi level. There are several sharp states at defined energies, which increase in number and intensity as the conductor length *L* is increased. These states that appear in the energy range corresponding to the gap of a pristine *N *= 5 A-GNRs [24,32] correspond to a constructive interference of the electron wavefunctions inside the heterostructure, which can travel forth and back generating stationary (well-like) states. In this sense, the finite length of the central ribbons imposes an extra spatial confinement to electrons, as analogy of what happens in open quantum dot systems [16,17,19,33,34]. Independently of their sharp line shape, these discrete levels behave as resonances in the system allowing the conduction of electrons at these energies, as it is shown in the corresponding conductance curves of Figure 2b. It is clear that as the conductor length is increased, the number of conductance peaks around the Fermi level is also increased, tending to form a plateau of one quantum of conductance (*G*_0 _= 2*e*^2^/*h*) at this energy range. These conductance peaks could be modulated by the external perturbations, as we will show further in this work.

At higher energies, the conductance plateaus appear each as 2*G*_0_, which is explained by the definition of the transmission probability *T*(*E*) of an electron passing through the conductor. In these types of heterostructures, if the conductor is symmetric (*N*_*u *_=*N*_*d*_), the number of allowed transverse channels are duplicated; therefore, electrons can be conduced with the same probability through both finite ribbons. On the other hand, in Figure 2c, we present results of conductance for a conductor of length *L *= 15 and composed of two A-GNRs of widths *N*_*d *_= 5 and *N*_*u *_= 7, connected to two leads of widths *N *= 17. As a comparison, we have included the corresponding pristine cases. As it is expected, the conductance for an asymmetric configuration (red curve) reflects the exact addition of the transverse channels of the constituent ribbons, with the consequent enhancement of the conductance of the systems. Nevertheless, there is still only one quantum of conductance near the Fermi energy due to the resonant states of the finite system, whether the constituent ribbons are semiconductor or semimetal. We have obtained these behaviours for different configurations of conductor, considering variations in length and widths of the finite ribbons and leads.

### Magnetic field effects

**Figure 3:**
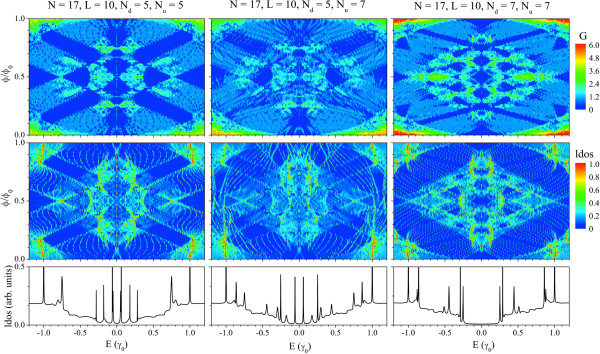
**Magnetic field effects on LDOS and conductance.** Contour plots of LDOS (lower panels) and conductance (upper panels) as a function of the Fermi energy and the magnetic flux crossing the hexagonal lattice for three different configurations of conductor. As a comparison, we have included the LDOS curves of the corresponding system without the magnetic field (bottom plots).

In what follows, we will include the interaction of a uniform external magnetic field applied perpendicularly to the conductor region. We have considered in our calculations that the magnetic field could affect the ends of the leads, forming an effective ring of conductor. The results of LDOS and conductance as a function of the Fermi energy and the normalized magnetic flux (*ϕ*/*ϕ*_0_) for three different conductor configurations are displayed in the contour plots of Figure 3. The left panels correspond to a symmetric system composed of two metallic A-GNRs of widths *N*_*u *_=*N*_*d *_= 5. The central panels correspond to an asymmetric conductor composed of two A-GNRs of widths *N*_*d *_= 5 (metallic) and *N*_*u *_= 7 (semiconductor). The right panels correspond to a symmetric system composed of two semiconductor A-GNRs of widths *N*_*u *_=*N*_*d *_= 7. All configurations have been considered of the same length *L *= 10 and connected to the same leads of widths *N *= 17. Finally, we have included as a reference, the plots of LDOS versus Fermi energy for the three configurations.

From the observation of these plots, it is clear that the magnetic field strongly affects the electronic and transport properties of the considered heterostructures, defining and modelling the electrical response of the conductor. In this sense, we have observed that in all considered systems, periodic metal-semiconductor electronic transitions for different values of magnetic flux ratio *ϕ*/*ϕ*_0_, which are qualitatively in agreement with the experimental reports of similar heterosructures [21-23]. Although the periodic electronic transitions are more evident in symmetric heterostructures (left and right panels), it is possible to obtain a similar effect in the asymmetric configurations. These behaviours are direct consequences of the quantum interference of the electronic wave function inside this kind of annular conductors, which in general present an Aharonov-Bohm period as a function of the magnetic flux.

The evolution of the electronic levels of the system, depending of their energy, exhibits a rich variety of behaviours as a function of the external field. In all considered cases, the LDOS curves exhibit electronic states pinned at the Fermi Level, at certain magnetic flux values. This state corresponds to a non-dispersive band, equivalent with the supersymmetric Landau level of the infinite two-dimensional graphene crystal [30,35]. At low energy region and for low magnetic field, it is possible to observe the typical square-root evolution of the relativistic Landau levels [36]. The electronic levels at highest energies of the system evolve linearly with the magnetic flux, like regular Landau levels. This kind of evolution is originated by the massive bands in graphene, which is expected for these kinds of states in graphene-based systems [37,38].

By comparing the LDOS curves and the corresponding conductance curves, it is possible to understand and define which states contribute to the transport of the systems (resonant tunneling peaks), and which ones only evolve with the magnetic flux but remain as localized states (quasi-bond states) of the conductor. These kind of behaviour has been reported before in similar systems [19,20]. This fact is more evident in the symmetric cases, where there are several states in the ranges *ϕ*/*ϕ*_0_∈[0.1,0.9] and *E*(*γ*_0_)∈[−1.0,1.0] of the LDOS curves which evolve linearly with the magnetic flux, but are not reflected in the conductance curves. In fact, at these ranges, the conductance curves exhibit marked gaps with linear evolution as a function of the magnetic flux. For the asymmetric case, it is more difficult to define which states behave similarly; however, there are still some regions at which the conductance exhibits gaps with linear evolution as a function of the magnetic flux. All these electronic modulations could be useful to generate on/off switches in electronic devices, by changing in a controlled way the magnetic field intensity applied to the heterostructures. We have obtained these behaviours for different configurations of conductor, considering variations in length and widths of the finite ribbons and leads.

## Conclusions

In this work, we have analysed the electronic and transport properties of a conductor composed of two parallel and finite A-GNRs, connected to two semi-infinite lead, in the presence of an external perturbation. We have thought these systems as two parallel wires of an hypothetical circuit made of graphene, and we have studied the transport properties as a function of the separation and the geometry of these ‘wires’, considering the isolated case and the presence of an external magnetic field applied to the system. We have observed resonant tunneling behaviour as a function of the geometrical confinement and a complete Aharonov-Bohm type of modulation as a function of the magnetic flux. These two behaviours are observed even when the two A-GNRs have different widths, and consequently, different transverse electronic states. Besides, the magnetic field generates a periodic metal-semiconductor transition of the conductor, which can be used in electronics applications. We want to note that our results are valid only in low temperature limits and in the absence of strong disorder into the systems. In the case of non-zero temperature, it is expected that the resonances in the conductance curves will become broad and will gradually vanish at room temperature [20].

## Competing interests

The authors declare that they have no competing interests.

## Authors’ contributions

LR and JWG have worked equally in all results presented in this paper. Both authors read and approved the final manuscript.

## Authors’ information

LR is a professor at the Physics Department, Technical University Federico Santa Maria, Valparaiso, Chile. JWG is a postdoctoral researcher at the International Iberian Nanotechnology Laboratory, Braga, Portugal.
